# Multimodality imaging to identify lipid-rich coronary plaques and predict periprocedural myocardial injury: Association between near-infrared spectroscopy and coronary computed tomography angiography

**DOI:** 10.3389/fcvm.2023.1127121

**Published:** 2023-03-30

**Authors:** Hideaki Ota, Hitoshi Matsuo, Shunsuke Imai, Yuki Nakashima, Yoshiaki Kawase, Munenori Okubo, Hiroshi Takahashi, Hideki Kawai, Yoshihiro Sobue, Masanori Kawasaki, Takeshi Kondo, Takashi Muramatsu, Hideo Izawa

**Affiliations:** ^1^ Department of Cardiology, Gifu Heart Center, Gifu, Japan; ^2^ Department of Cardiology, Fujita Health University Hospital, Toyoake, Japan; ^3^ Department of Radiology, Gifu Heart Center, Gifu, Japan; ^4^ Department of Cardiology, Fujita Health University Bantane Hospital, Nagoya, Japan

**Keywords:** near-infrared spectroscopy, coronary computed tomography angiography, lipid-rich plaque, percutaneous coronary intervention, periprocedural myocardial injury (PMI)

## Abstract

**Background:**

This study compares the efficacy of coronary computed tomography angiography (CCTA) and near-infrared spectroscopy intravascular ultrasound (NIRS–IVUS) in patients with significant coronary stenosis for predicting periprocedural myocardial injury during percutaneous coronary intervention (PCI).

**Methods:**

We prospectively enrolled 107 patients who underwent CCTA before PCI and performed NIRS–IVUS during PCI. Based on the maximal lipid core burden index for any 4-mm longitudinal segments (maxLCBI4mm) in the culprit lesion, we divided the patients into two groups: lipid-rich plaque (LRP) group (maxLCBI4mm ≥ 400; *n* = 48) and no-LRP group (maxLCBI4mm < 400; *n* = 59). Periprocedural myocardial injury was a postprocedural cardiac troponin T (cTnT) elevation of ≥5 times the upper limit of normal.

**Results:**

The LRP group had a significantly higher cTnT (*p* = 0.026), lower CT density (*p* < 0.001), larger percentage atheroma volume (PAV) by NIRS–IVUS (*p* = 0.036), and larger remodeling index measured by both CCTA (*p* = 0.020) and NIRS–IVUS (*p* < 0.001). A significant negative linear correlation was found between maxLCBI4mm and CT density (rho = −0.552, *p* < 0.001). Multivariable logistic regression analysis identified maxLCBI4mm [odds ratio (OR): 1.006, *p* = 0.003] and PAV (OR: 1.125, *p* = 0.014) as independent predictors of periprocedural myocardial injury, while CT density was not an independent predictor (OR: 0.991, *p* = 0.22).

**Conclusion:**

CCTA and NIRS–IVUS correlated well to identify LRP in culprit lesions. However, NIRS–IVUS was more competent in predicting the risk of periprocedural myocardial injury.

## Introduction

Acute coronary syndrome (ACS) occur in rupture of lipid-rich plaques (LRPs). Additionally, LRP is associated with distal slow flow or no reflow during percutaneous coronary intervention (PCI), resulted in the periprocedural myocardial injury (1). Moreover, those complications, as defined by elevated myocardial troponin levels, are associated with a poor prognosis after PCI (2).

Coronary computed tomography angiography (CCTA) has allowed for non-invasive assessment of coronary artery disease (CAD) and plaque morphology (3, 4). Some of these findings derived CCTA, including low CT density value, positive vessel remodeling, spotty calcification, and signet ring-like enhancement, are associated with plaque instability (4, 5).

As a novel imaging method for the detection of LRPs, near-infrared spectroscopy intravascular ultrasound (NIRS-IVUS) has recently been emerged (6, 7). Moreover, the prospective studies revealed the relationship between major adverse cardiovascular events (MACE) and plaque lipid component in the non-culprit lesions as assessed by NIRS-IVUS (8, 9).

CCTA and NIRS-IVUS could access for detecting “vulnerable” plaque in a different way. However, it is unclear which one provides more accurate prediction of periprocedural myocardial injury. This study assesses the relationship between CCTA features and NIRS-derived plaque characteristics in patients with CAD and investigates the association between plaque characteristics derived from both modalities in patients with periprocedural myocardial injuries.

## Methods

### Study population

Patients with CAD and clinically relevant coronary artery stenosis assessed by CCTA at Gifu Heart Center (Gifu, Japan) were screened from October 2016 to January 2020. In addition, patients who were diagnosed with non-ST-elevation ACS (NSTE-ACS), stable angina pectoris (SAP), and silent myocardial ischemia (SMI) and scheduled for PCI with NIRS–IVUS (LipiScan, InfraReDx, Burlington, MA, USA) within 90 days after CCTA were prospectively enrolled. Detailed diagnosis definitions are given in the Supplementary Methods. We decided the target lesions according to the evidence of myocardial ischemia by electrocardiogram (ECG), echocardiographic, and physiological findings such as fractional flow reserve (FFR) or stress myocardial perfusion imaging before PCI in correspondence with the most narrowing lesion on coronary angiogram (CAG). The lesion with FFR < 0.80 was diagnosed as positive functional ischemia and considered for PCI.

Patient-based exclusion criteria were (1) simultaneous treatment with multivessel PCI at the initial procedure, (2) chronic kidney disease manifested by an estimated glomerular filtration rate of <30 ml/min/1.73 m^2^, (3) presentation with hemodynamic instability, and (4) severe valvular heart diseases. Lesion-based exclusion criteria were (1) lesions that have already been stented before and those undergoing stent placement during the index PCI, including 5-mm proximal/distal borders; (2) lesions belonging to a vessel that had undergone coronary artery bypass grafting; (3) left main trunk lesions; (4) ostial lesions (<10 mm from the left main bifurcation or coronary ostia to the lesion edge); (5) long lesions requiring two or more stents per lesion; (6) severe calcified lesions requiring atheroablative device prior to NIRS-IVUS imaging; (7) severe stenotic lesions for which difficulty was anticipated in advancing the NIRS-IVUS catheter without plain balloon angioplasty of ≥2.0-mm diameter; (8) poor image quality for which the technician could not perform either CCTA or NIRS-IVUS analyses; (9) lesions located in a small vessel [with an external elastic membrane (EEM) diameter of <2.0 mm]; and (10) use of a distal protection device during PCI.

In accordance with the above criteria, we enrolled 142 consecutive patients in the study after CCTA. Of these, we excluded 17 patients who met patient exclusion the criteria before undergoing PCI and 11 patients who met the lesion exclusion criteria at the time of PCI. Additionally, 5 patients who were unable to provide informed consent and 2 patients who did not undergo PCI were excluded. Finally, we included 107 patients (98 with SAP/SMI and 9 with NSTE-ACS) in the study.

Written informed consent was given by each patient. The study was conducted in accordance with the Declaration of Helsinki and authorized by the Institutional Review Board (2015015). This study was registered with the University Medical Information Network Clinical Trial Registry (UMIN000042883).

### CCTA measurement protocol

The CCTA protocol is provided in the Supplementary Methods. Briefly, ECG-gated CT angiography was performed using SOMATOM Definition AS+ (Siemens Healthcare, Fordchheim, Germany). Iopamiron 370 mg iodine/ml (Bayer Health Care, Osaka, Japan) or Omnipaque 350 mg iodine/ml (Daiichi Sankyo, Tokyo, Japan) were used in a contrast-enhanced ECG gating scan to collect CCTA data.

Experienced technicians blinded to quantitative coronary angiography (QCA) and NIRS–IVUS performed the scan analysis using the Aquarius iNtuition edition version 4.4.8 (TeraRecon, Frankfurt, Germany) three-dimensional workstation. Cross-sectional images were used to evaluate the outer vessel area and the remodeling index (RI) of the vessel. The RI was calculated as the EEM cross-sectional area (CSA) of the target lesion divided by the average of the EEM-CSAs of the proximal and distal reference segments; positive remodeling was defined as a RI of ≥1.10 (4).

We evaluated the following plaque characteristics: (1) CT density, (2) spotty calcification, and (3) napkin-ring sign. The CT Hounsfield unit (HU) values of the culprit plaque were determined from at least three rounded regions of interest of 0.5 mm^2^, and the unit score at each region of interest was measured and then averaged. Low-attenuated plaque was defined as any voxel of <30 HU within a coronary plaque (10). Spotty calcification was classified as <3 mm in size on curved multiplanar reformation images and unilateral on cross-sectional images (4). The following criteria were used to define a napkin-ring sign: (1) existence of high attenuation ring around certain coronary artery plaques or (2) CT attenuation of the ring, presenting higher than those of the adjacent plaque and not >130 HU (11).

### Blood cardiac marker sampling protocol

In this study, cTnT was detected by HISCL™ Troponin-T hs assay kit (Sysmex, Kobe, Japan), with a suggested upper limit of normal (ULN) cutoff value of 0.016 ng/ml; thus, periprocedural myocardial injury was defined as a cTnT level of >5 times the ULN at either 6 or 18–24 h after PCI, according to the fourth universal definition of MI (12). In patients with NSTE-ACS who had elevated preprocedural cTnT levels, the postprocedural cTnT must rise by >20% from the preprocedural value. However, the absolute postprocedural value must still be >5 times our institution's ULN.

### Coronary angiography, PCI, and NIRS–IVUS image acquisition procedure

The Supplementary Methods provide a description of the preoperative antiplatelet therapy and perioperative anticoagulants. CAG and PCI were performed using standard techniques, and the selection of devices and pharmacotherapy was at the operator's discretion.

Following intracoronary injection of 2 mg of isosorbide dinitrate, NIRS–IVUS images were obtained using a commercially available system (LipiScan, InfraReDx, Burlington, MA, USA). The NIRS-IVUS system used a 40-MHz (TVC Insight™, model TVC-MC8) or 50-MHz transducer (Dualpro™, model TVC-MC10) in a 3.2 Fr catheter. NIRS–IVUS imaging was performed before either direct stent implantation or balloon predilatation. An automatic mechanical pullback was used to bring the IVUS catheter back to the aorto-ostial junction at a speed of 0.5 mm/s. The system demonstrates grayscale IVUS images and color-coded NIRS data in a single pullback at the same time, as described in Supplementary Methods. All image data were stored on a DVD and forwarded to the local core laboratory for analysis.

A verified automated edge-detection software (CCIP-310/W, Cathex, Tokyo, Japan) was used to perform QCA analysis. Using orthogonal projections, the minimum lumen diameter, reference diameter, and lesion length were assessed in diastolic frames.

### Gray-scale IVUS and NIRS analyses and chemometrics

Off-line gray-scale IVUS and NIRS analyses were performed using QIvus 3.1 (Medis Medical Imaging Systems, Leiden, The Netherlands). Experienced investigators blinded to the clinical findings, angiography, and CCTA characteristics analyzed these data.

On the basis of the American College of Cardiology Clinical Expert Consensus Document on Standards for Acquisition, Measurement, and Reporting of IVUS Investigations, whole lesion segments were cross-analyzed (13).

Measurements of the regions containing the minimal luminal area (MLA) were acquired every 0.5 mm. The slice with the largest lumens within 10 mm of the MLA site and prior to any significant side branches was referred to as the proximal or distal reference segment. These measurements included EEM CSA and lumen CSA. EEM CSA minus lumen CSA was used to determine plaque plus media CSA, and percent plaque burden (PB) was calculated as (plaque plus media CSA/EEM CSA) × 100. The average area of the proximal/distal reference segment EEM CSAs was divided by the EEM CSA at the MLA site to determine the RI.

Vessel, lumen, and plaque volume analysis was calculated using Simpson's rule. Normalized total atheroma volume (TAV) was estimated using the following formula (14):

Normalized TAV = [Σ (EEM CSA−Lumen CSA)/Number of images in pullback] × median number of images in cohort.

In order to account for analysis length, the other volumetric parameters were also normalized.

Percent atheroma volume (PAV) was calculated according to this equation:

$$\mathrm{PAV}(\text{\%})=\left[\frac{\Sigma \;(\mathrm{EEM}\;\mathrm{CSA}-\mathrm{Lumen}\;\mathrm{CSA})}{\Sigma \mathrm{EEM}\;\mathrm{CSA}}\right]\times 100.$$

Gray-scale IVUS derived attenuated plaque was defined as IVUS images with backward signal attenuation of ≥180 degree behind plaque without dense calcium (15). The maxLCBI4mm was calculated as the maximum value of the lipid-core burden index (LCBI) for any 4-mm segment in the target lesion, including the MLA (Supplementary Methods).

**Figure 1:**
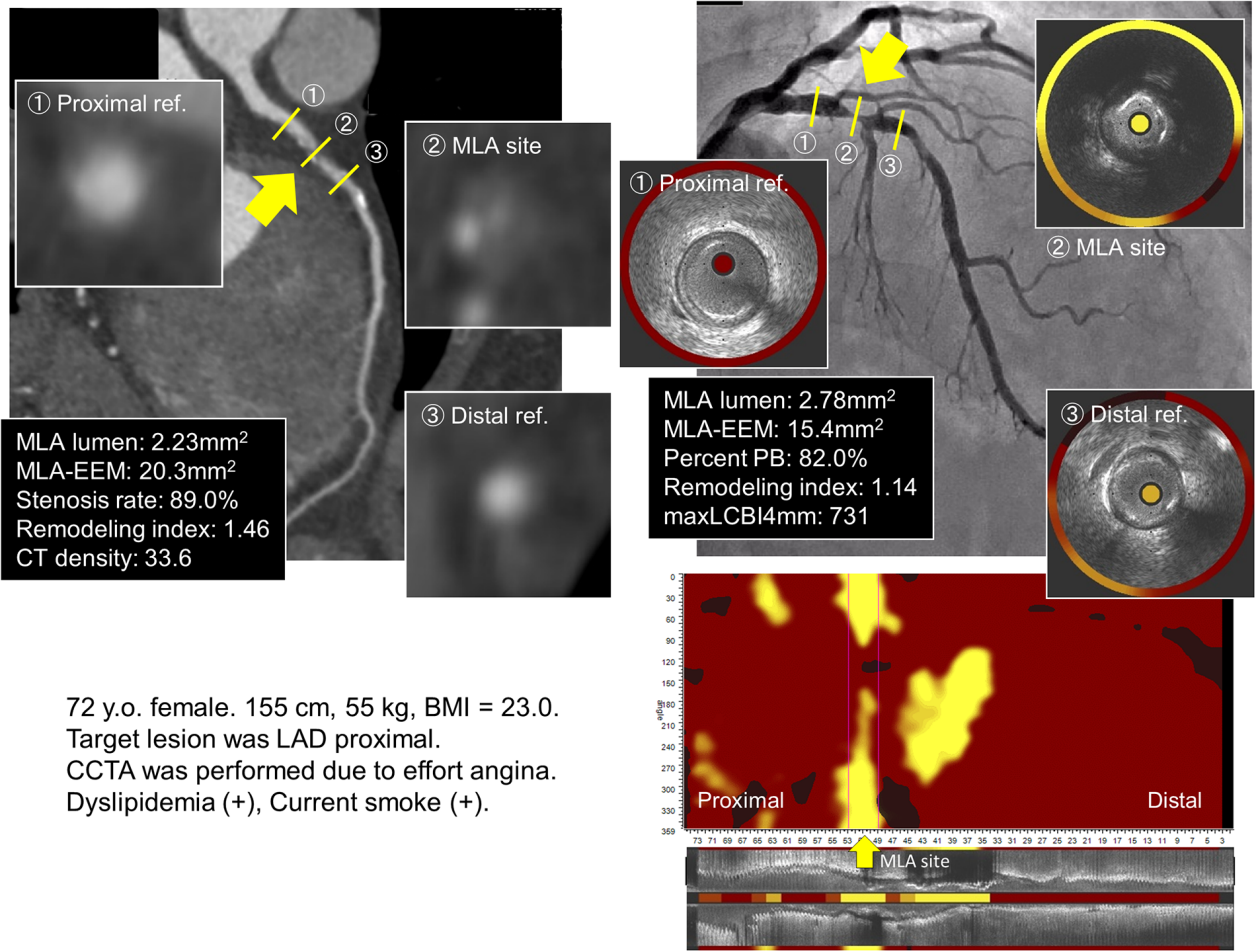
Coronary angiography, CCTA, gray-scale IVUS, and NIRS for the lipid-rich plaque group. The culprit lesion is located at the proximal LAD. CCTA revealed low CT density with positive remodeling; similarly, NIRS–IVUS showed high maxLCBI4mm with positive remodeling in the culprit segment. (The yellow arrow indicates the site of the minimal lumen area.) The cTnT level increased from 0.002 ng/ml to 0.124 ng/ml after PCI. CCTA, coronary computed tomography angiography; IVUS, intravascular ultrasound; NIRS, near-infrared spectroscopy; LAD, left anterior descending; maxLCBI4mm, maximum value of the lipid-core burden index for any 4-mm segment; cTnT, cardiac troponin T; PCI, percutaneous coronary intervention.

Figure 1 presents an example of the patient who underwent CCTA and NIRS-IVUS.

### Statistical analysis

The normality of the variables was assessed using the Shapiro–Wilk test. Continuous variables were compared using the Student's *t*-test or the Mann–Whitney *U* test, and were presented as mean ± SD or median and interquartile range (IQR). Chi-squared or Fisher's exact test was used to compare categorical variables that were presented as numbers and percentages. Differences in cTnT from the baseline value within each group were analyzed using Friedman test, and Bonferroni correction was added for post-hoc analysis. Spearman's correlation was utilized to analyze the relationship between the CCTA–measured parameters and the NIRS–IVUS–derived parameters. Inter- and intra-observer reproducibility on CCTA- and IVUS parameters were assessed using intraclass correlation coefficient. The interobserver reproducibility for CT density and maxLCBI4mm were 0.81 and 0.90 as Kappa coefficients, respectively. Moreover, the intraobserver Kappa coefficients for CT density and maxLCBI4mm were 0.86 and 0.92, respectively.

Multivariable logistic regression analysis was utilized to evaluate the relationship between periprocedural myocardial injury and clinical characteristics, angiographic characteristics, CCTA/NIRS-IVUS findings, and other potential confounders. Given the limited number of subclinical cTnT in this study, variables with *p* < 0.05 in the univariable analysis and clinically meaningful associations with periprocedural myocardial injury were included in the multivariable logistic regression model. To determine the best cutoff threshold of the independent risk factors for incidence of periprocedural myocardial injury, the receiver-operating characteristic (ROC) analysis was calculated. In the present study, the Youden Index was defined as the best cut-off threshold.

The statistical significance level was established at *p* < 0.05. All statistical analyses were performed using IBM SPSS version 28.0 (IBM Corp., Armonk, NY, United States).

## Results

### Baseline characteristics and procedural findings

**Figure 2:**
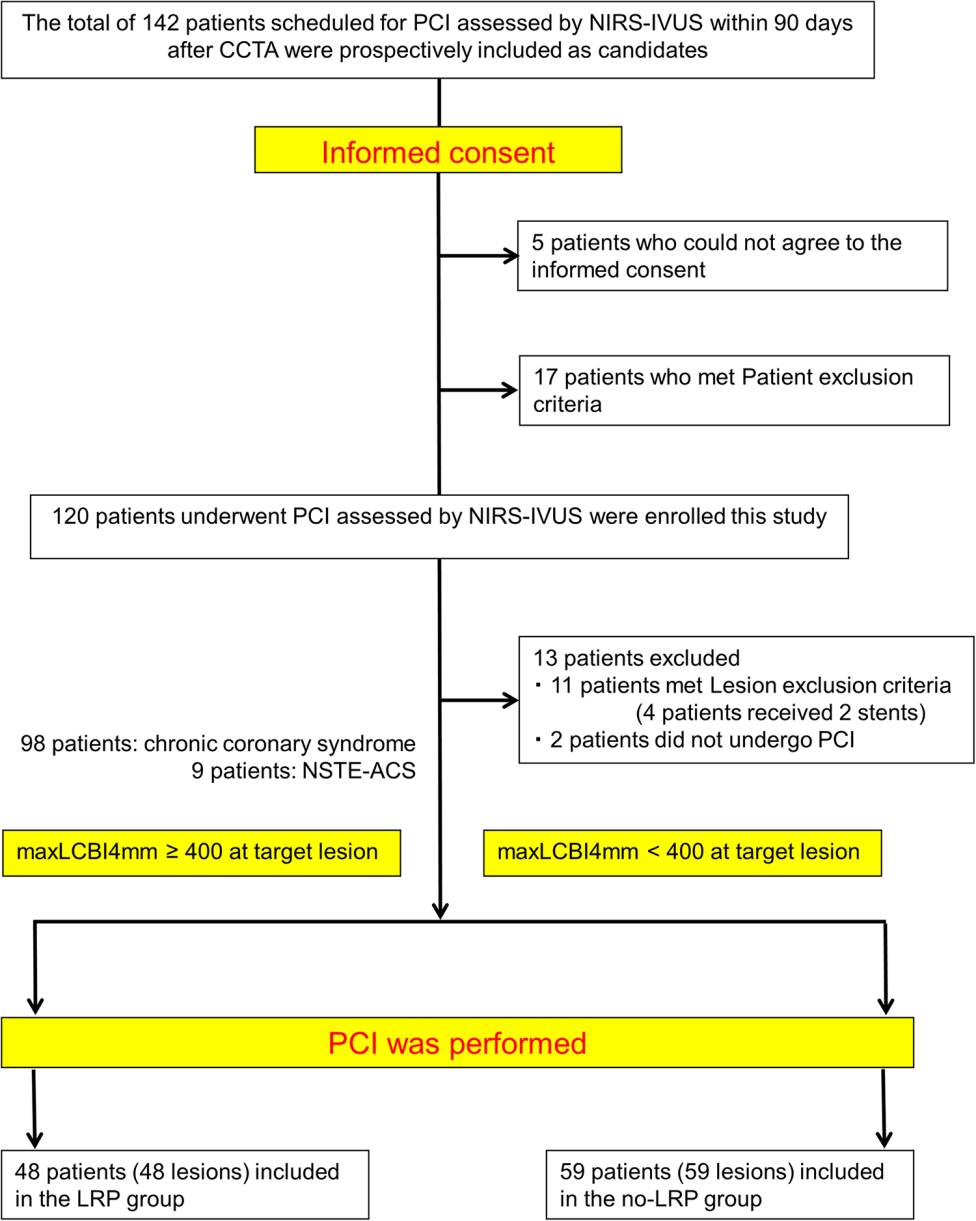
Study flow chart. PCI, percutaneous coronary intervention; NIRS, near-infrared spectroscopy; IVUS, intravascular ultrasound; CCTA, coronary computed tomography angiography; NSTE-ACS, non–ST-elevation acute coronary syndrome; maxLCBI4mm, maximal lipid core burden index at the 4-mm segment; LRP, lipid-rich plaque.

A total of 107 patients were divided into two groups according to the maxLCBI4mm, using 400 as the cutoff value based on previous investigation (Figure 2) (8, 16). Of these patients, 48 (45.8%) were allocated to the LRP group (maxLCBI4mm ≥ 400) and 59 (54.2%) to the no-LRP group (maxLCBI4mm < 400).

Baseline characteristics and procedural findings are shown in Table 1. Between the groups, there were no significant differences in patient characteristics except for percentage of angiotensin-converting enzyme inhibitor/angiotensin II receptor blocker. Otherwise, larger number of patients with left anterior descending lesion was included in the LRP group according to the baseline lesion characteristics.

**Table 1:** Baseline patient and lesion characteristics.

	LRP (*n* = 48)	No-LRP (*n* = 59)	*p*-value
***Demographics and risk factors***
Age (years)	69.8 ± 9.7	66.7 ± 10.5	0.12
Male	35 (72.9)	47 (79.7)	0.41
Body mass index	23.9 ± 2.9	25.1 ± 3.3	0.059
Hypertension	40 (83.3)	49 (83.1)	0.97
Dyslipidemia	37 (77.1)	45 (76.3)	0.92
Diabetes mellitus	11 (22.9)	23 (39.0)	0.076
Smoking	31 (64.6)	46 (78.0)	0.13
Current smoker	13 (27.1)	22 (37.3)	0.24
Estimated GFR	65.6 ± 14.5	69.8 ± 15.2	0.14
CKD (estimated GFR ≤ 60 ml/min/1.73 m^2^)	15 (31.3)	15 (25.4)	0.50
**Past medical history**
Myocardial infarction	7 (14.6)	7 (11.9)	0.68
Percutaneous coronary intervention	16 (33.3)	17 (28.8)	0.61
Family history of coronary artery disease	11 (22.9)	19 (32.2)	0.28
Ejection fraction (%)	62.2 ± 10.0	62.2 ± 8.5	0.99
***Biomarkers before PCI***
**Lipid profiles (mg/dl)**
Total cholesterol	163.2 ± 33.3	156.7 ± 36.0	0.34
LDL-C	93.2 ± 30.1	88.3 ± 27.9	0.38
HDL-C	47.9 ± 10.0	47.5 ± 12.0	0.86
Triglyceride	130.5 (82.3–194.3)	117.0 (85.5–168.5)	0.40
Hemoglobin A1c (%)	5.8 (5.6–6.1)	5.9 (5.7–6.5)	0.39
High-sensitivity C-reactive protein (mg/dl)	0.07 (0.05–0.18)	0.07 (0.04–0.17)	0.57
***Drug medications before PCI***
Beta-blocker	26 (54.2)	28 (47.5)	0.49
Calcium antagonist	28 (58.3)	35 (59.3)	0.92
ACEI/ARB	38 (79.2)	33 (55.9)	0.011
Statin	42 (87.5)	50 (84.7)	0.68
Insulin	0 (0.0)	2 (3.4)	0.20
Diabetes oral drug	7 (14.6)	13 (22.0)	0.33
***Clinical diagnosis***			0.89
Silent myocardial ischemia/stable angina	44 (91.7)	54 (91.5)	
NSTE-ACS
Unstable angina	1 (2.1)	2 (3.4)	
NSTEMI	3 (6.3)	3 (5.1)	
***Target vessel***			0.008
Left anterior descending	39 (79.6)	34 (58.6)	
Left circumflex	8 (16.3)	9 (15.5)	
Right coronary artery	2 (4.1)	15 (25.9)	
***Target lesion location***			0.050
Proximal	16 (33.3)	27 (46.6)	
Mid	30 (62.5)	24 (41.4)	
Distal	2′ (4.2)	8 (13.8)	
***PCI procedural data***
Implanted stent diameter (mm)	3.00 (2.75–3.00)	3.00 (2.75–3.00)	0.35
Implanted stent length (mm)	26.0 (20.0–33.3)	23.0 (18.0–28.0)	0.043
Stent deployment pressure (atm)	12.0 (12.0–12.5)	12.0 (11.0–13.0)	0.21
Post dilatation	41 (85.4)	48 (81.4)	0.58

Values are mean ± SD, median (interquartile range), or *n* (%).

LRP, lipid-rich plaque; eGFR, estimated glomerular filtration rate; CKD, chronic kidney disease; PCI, percutaneous coronary intervention; LDL-C, low-density lipoprotein cholesterol; HDL-C, high-density lipoprotein cholesterol; ACEI, angiotensin-converting-enzyme inhibitor; ARB, angiotensin II receptor blocker; NSTEMI, non-ST elevation myocardial infarction.

eGFR was calculated using the following formula for Japanese patients: eGFR (male) = 194 × serum creatinine^−1.094^ × age ^−0.287^, and eGFR (female) = (194 × serum creatinine^−1.094^ × age^−0.287^) × 0.739.

Hypertension is defined as a patient with blood pressure >140/90 mmHg or on medication.

Dyslipidemia is defined as a patient with fasting cholesterol >250 mg/dl or LDL-cholesterol >140 mg/dl or on medication.

CKD is defined as a patient with eGFR <60 ml/min/1.73 m^2^.

In terms of procedural findings, the LRP group received significantly longer stents (*p* = 0.043), whereas no significant differences in other parameters were observed between the groups.

### Biomarker profiles during PCI

**Figure 3:**
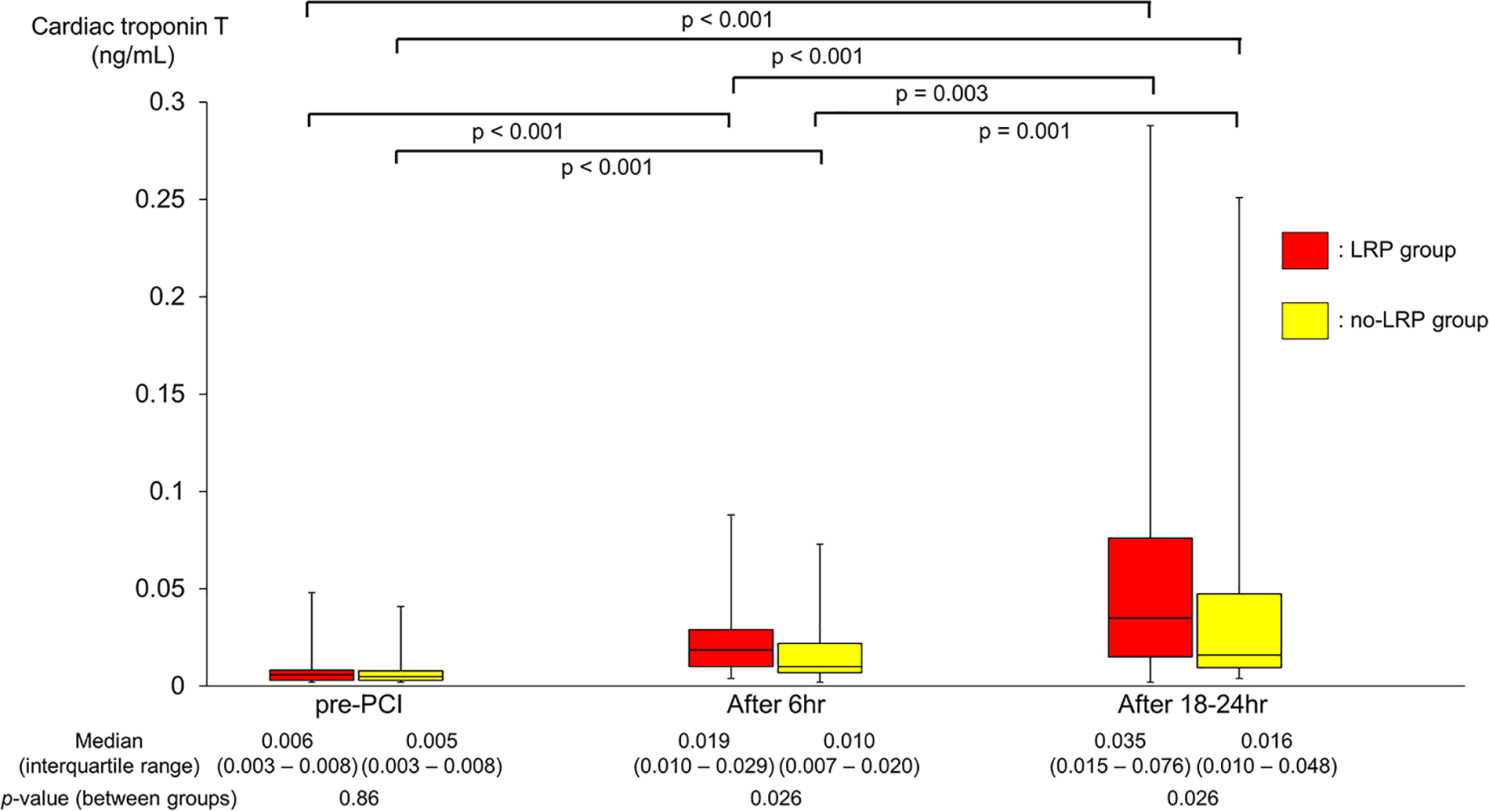
Changes in cTnT levels during PCI. cTnT, cardiac troponin T; PCI, percutaneous coronary intervention; LRP, lipid-rich plaque.

Comparable baseline biomarker levels including cTnT (0.006 vs. 0.005 ng/ml; *p* = 0.86) were found for both groups (Table 1 and Figure 3). However, during the study period, the LRP group showed a significantly greater and faster increase in cTnT levels (0.019 vs. 0.010 ng/ml at 6 h, 0.035 vs. 0.016 ng/ml at 18–24 h; *p* = 0.026 for both). Consequently, there was a significantly higher proportion of periprocedural myocardial injury in the LRP group (13 patients [27.1%] vs. 6 patients [10.2%]; *p* = 0.023).

### QCA and CCTA parameters at baseline PCI

QCA and CCTA measurements are summarized in Table 2. The LRP group was characterized by larger RI and lower CT density than those in the no-LRP group (1.30 vs. 1.18, *p* = 0.020; 52.9 vs. 96.7, *p* < 0.001, respectively). Moreover, the prevalence of positive remodeling and low attenuated plaque were significantly greater in the LRP group than in the no-LRP group (87.5% vs. 66.1%, *p* = 0.010; 22.9% vs. 4.1%, *p* = 0.002, respectively). In terms of CCTA-derived plaque morphology, the LRP group had a higher rate of lesions with the napkin-ring sign (50.0% vs. 28.8%; *p* = 0.025).

**Table 2:** QCA and CCTA analysis at baseline.

	LRP (*n* = 48)	No-LRP (*n* = 59)	*p*-value
***QCA***
Minimal lumen diameter (mm)	1.06 (0.91–1.27)	1.22 (0.92–1.46)	0.12
Reference diameter (mm)	2.64 (2.47–2.93)	2.81 (2.35–3.04)	0.80
Percent diameter stenosis (%)	59.0 (54.3–65.3)	54.0 (49.0–64.0)	0.055
Lesion length (mm)	21.9 (16.3–29.2)	18.9 (13.1–24.4)	0.058
Eccentric plaque (*n*, %)	23 (47.9)	38 (64.4)	0.087
Calcification (*n*, %)	5 (10.4)	5 (8.5)	0.73
Thrombus (*n*, %)	1 (2.1)	0 (0.0)	0.27
***CCTA***
**At minimal lumen site**
Lumen area (mm^2^)	2.39 (2.07–3.02)	2.59 (1.99–3.18)	0.65
Vessel area (mm^2^)	15.9 (12.0–20.4)	14.4 (11.4–19.3)	0.45
Plaque area (mm^2^)	13.3 (9.5–18.1)	12.4 (9.2–15.8)	0.40
Percent area stenosis (%)	84.0 (78.8–88.6)	82.3 (80.0–85.9)	0.24
Vessel area at reference site (mm2)	12.2 (10.2–14.5)	12.5 (10.2–14.8)	0.85
Remodeling index	1.30 (1.20–1.42)	1.18 (1.03–1.40)	0.020
Positive remodeling (RI >1.10)	42 (87.5)	39 (66.1)	0.010
CT density	52.9 (31.8–80.1)	96.7 (72.3–130.0)	<0.001
Low attenuated plaque (CT density <30 HU)	11 (22.9)	2 (4.1)	0.002
**CCTA-derived plaque morphology (*n*, %)**
Spotty calcification	23 (47.9)	27 (46.8)	0.82
Napkin-ring sign	24 (50.0)	17 (28.8)	0.025

Values are median (interquartile range) or *n* (%).

QCA, quantitative coronary angiography; CCTA, coronary computed tomography angiography; LRP, lipid-rich plaque.

### Gray-scale IVUS, NIRS parameters, and correlation of plaque morphology parameters with CCTA

Gray-scale IVUS and NIRS measurements are summarized in Table 3. At the MLA site, although there was no difference among groups in terms of gray-scale IVUS-measured lumen, vessel, and plaque areas, the LRP group had a larger percent PB and RI at the MLA site (79.9% vs. 77.2%, *p* = 0.029; 1.06 vs. 1.00, *p* < 0.001, respectively). Additionally, the percentage of patients with IVUS-attenuated plaques was significantly higher in the LRP group (54.2% vs. 15.3%, *p* < 0.001). Volumetric analysis also showed that PAV was significantly greater in the LRP group (58.4% vs. 54.9%; *p* = 0.036).

**Table 3:** Gray-scale IVUS and NIRS measurements during PCI.

	LRP (*n* = 48)	No-LRP (*n* = 59)	*p*-value
***Gray-scale IVUS***
**Minimal lumen site (mm^2^)**
Lumen CSA	2.56 (2.16–2.88)	2.58 (2.18–3.54)	0.34
EEM CSA	13.1 (10.5–16.0)	12.8 (10.0–16.3)	0.55
Plaque plus media CSA	10.0 (8.3–13.2)	9.6 (7.4–12.8)	0.28
Percent plaque burden (%)	79.9 (75.8–83.3)	77.2 (70.4–81.2)	0.029
Stented length (mm)	26.4 (20.4–34.7)	23.5 (18.8–29.0)	0.077
EEM CSA at reference site (mm^2^)	12.5 (10.1–14.6)	13.0 (11.1–15.4)	0.27
Remodeling index	1.06 (1.04–1.10)	1.00 (0.91–1.03)	<0.001
IVUS-attenuated plaque	26 (54.2)	9 (15.3)	<0.001
**Volumetric analysis (mm^3^)**
Normalized lumen volume	122.8 (109.3–154.6)	141.8 (113.6–173.1)	0.11
Normalized EEM volume	308.4 (263.3–352.7)	328.7 (272.1–373.9)	0.23
Normalized TAV	184.3 (145.3–215.5)	181.7 (152.7–215.2)	0.91
PAV (%)	58.4 (55.2–64.2)	54.9 (47.7–62.6)	0.036
***NIRS-IVUS measurements***
maxLCBI4mm	473.0 (434.3–625.5)	223.0 (114.5–308.5)	<0.001

Values are median (interquartile range) or *n* (%).

IVUS, intravascular ultrasound; NIRS, near-infrared spectroscopy; PCI, percutaneous coronary intervention; LRP, lipid-rich plaque; CSA, cross-sectional area; EEM, external elastic membrane; TAV, total atheroma volume; PAV, percent atheroma volume; maxLCBI4mm, maximum value of the lipid core burden index for any 4-mm segment.

**Figure 4:**
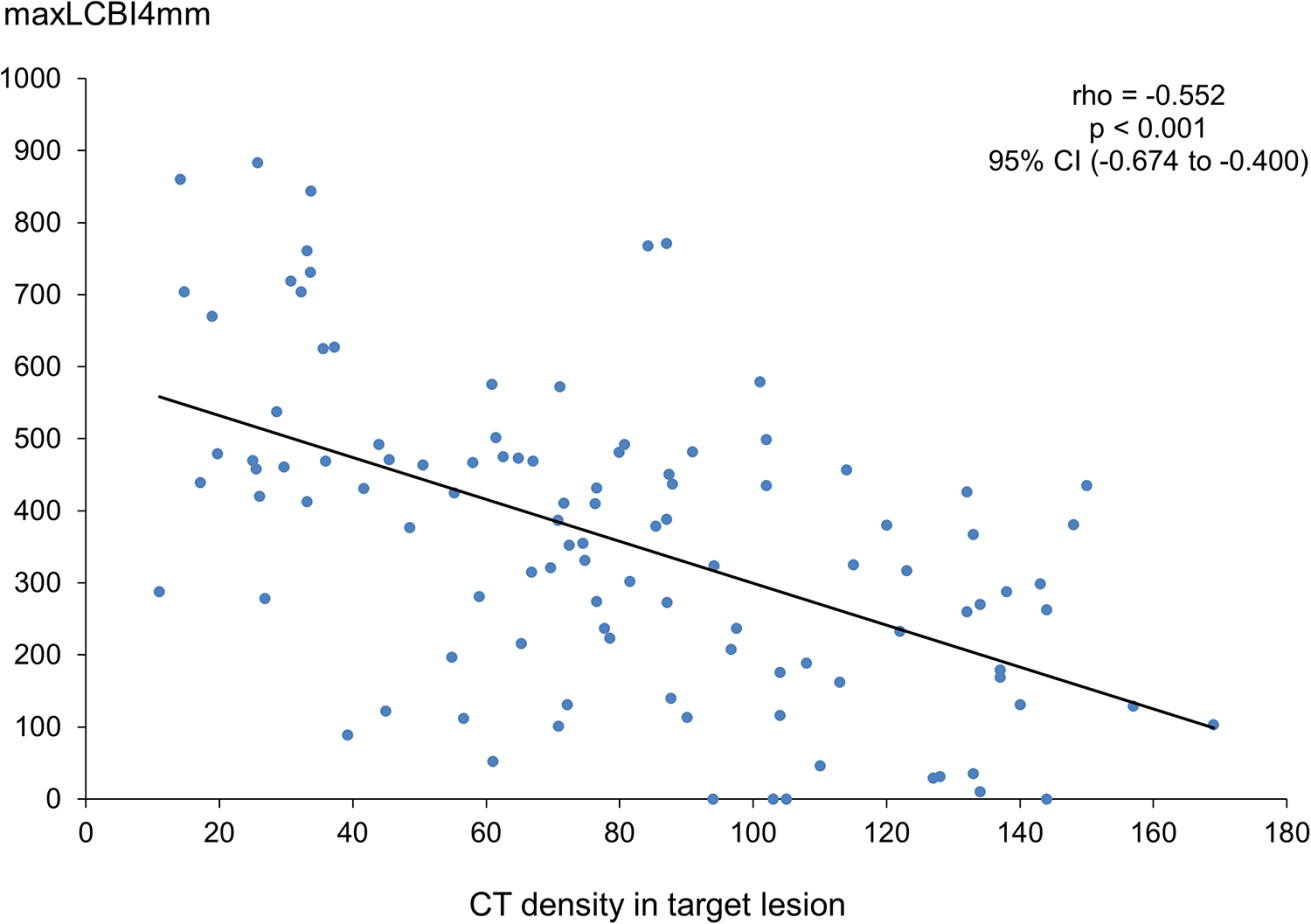
Correlation between CT density and maxLCBI4mm. CT, computed tomography; maxLCBI4mm, maximum value of the lipid-core burden index for any 4-mm segment; CI, confidence interval.

Figure 4 illustrates a significant negative linear correlation between CT density and maxLCBI4mm (rho = −0.552, *p *< 0.001).

### Predictors of periprocedural myocardial injury

Periprocedural myocardial injury was observed in 19 of the 107 patients (17.8%). According to the univariable analysis, QCA-measured lesion length, CT density, NIRS–IVUS-derived PAV, and maxLCBI4mm were significantly associated with periprocedural myocardial injury (Table 4). The strongest correlation between each of these modalities was between maxLCBI4mm and CT density, but there was also a significant correlation between the other factors, except for lesion length and CT density (Supplementary Table S1). Multivariable logistic regression analysis showed that the maxLCBI4mm (odds ratio [OR] 1.006, 95% confidence interval [CI] 1.002–1.011, *p* = 0.003) and PAV (OR 1.125, 95% CI, 1.025–1.235; *p* = 0.014) were independent predictors of periprocedural myocardial injury, whereas CT density was not an independent predictor (OR 1.010, 95% CI, 0.993–1.027; *p* = 0.25). CT density was not an independent predictor even when maxLCBI4mm was excluded from the model (OR 0.991, 95% CI, 0.977–1.005; *p* = 0.22).

**Table 4:** Analysis of factors related to periprocedural myocardial injury.

	Univariable Logistic Regression			Multivariable Logistic Regression					
				Model 1^a^			Model 2^b^		
	OR	95% CI	*p*-value	OR	95% CI	*p*-value	OR	95% CI	*p*-value
***Patient characteristics***
NSTE-ACS	0.556	0.065–4.726	0.59						
Diabetes mellitus	0.727	0.239–2.213	0.57						
Chronic kidney disease	1.231	0.420–3.606	0.71						
***Lesion characteristics***
Left anterior descending	0.759	0.269–2.139	0.60						
Proximal lesion	0.335	0.103–1.091	0.069						
***QCA***
Lesion length	1.065	1.003–1.132	0.041	1.039	0.967–1.116	0.30	1.046	0.979–1.118	0.18
***CCTA***
Remodeling index	2.346	0.303–18.163	0.41						
CT density	0.985	0.971–0.999	0.035	1.010	0.993–1.027	0.25	0.991	0.977–1.005	0.22
Napkin-ring sign	0.926	0.332–2.585	0.88						
***Gray-scale IVUS***
IVUS-attenuated plaque	2.800	1.017–7.709	0.046						
Normalized lumen volume	0.983	0.968–0.998	0.042						
Normalized TAV	1.007	0.999–1.015	0.082						
PAV	1.145	1.055–1.243	0.001	1.125	1.025–1.235	0.014	1.129	1.035–1.231	0.006
***NIRS-IVUS***
maxLCBI4mm	1.006	1.003–1.009	<0.001	1.006	1.002–1.011	0.003			

OR, odds ratio; CI, confidence interval; QCA, quantitative coronary angiography; NSTE-ACS, non-ST elevation acute coronary syndrome; QCA, quantitative coronary angiography; CCTA, coronary computed tomography angiography; IVUS, intravascular ultrasound; TAV, total atheroma volume; PAV, percent atheroma volume; NIRS, near-infrared spectroscopy; maxLCBI4mm, maximum value of the lipid core burden index for any 4-mm segment.

^a^ Model 1: Both CT density and maxLCBI4mm were added to lesion length and PAV.

^b^ Model 2: CT density was added to lesion length and PAV.

**Figure 5:**
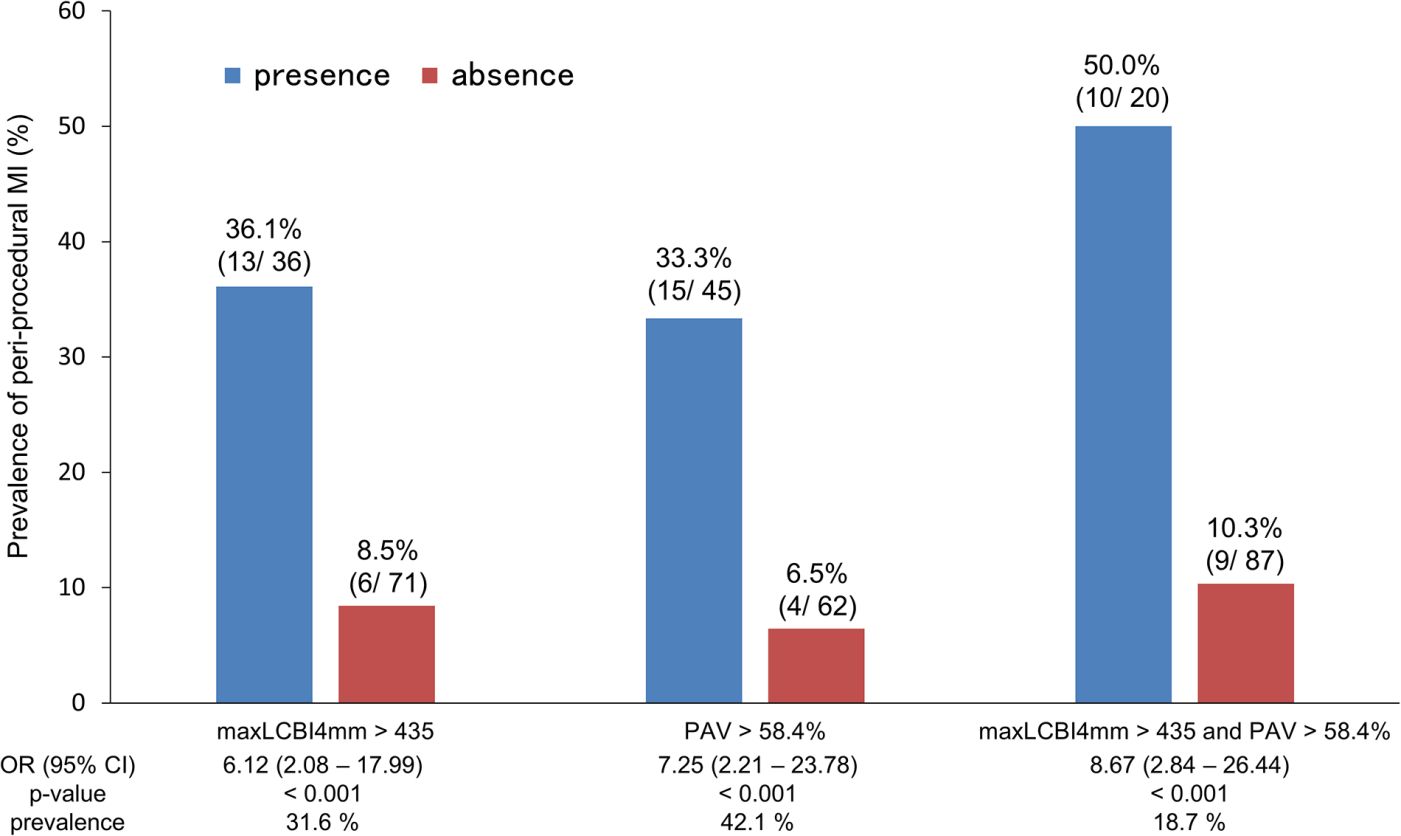
Frequency of periprocedural myocardial injury according to PAV and maxLCBI4mm. PAV, percent atheroma volume; maxLCBI4mm, maximum value of the lipid-core burden index for any 4-mm segment; OR, odds ratio; CI, confidence interval.

We compared the incidence of periprocedural myocardial injury by classifying each case according to the maxLCBI4mm of 435 and the PAV of 58.4%, calculated by the ROC analysis [area under the curve (AUC) 0.751, sensitivity 68.4%, specificity 76.1%; AUC 0.763, sensitivity 78.9%, specificity 67.0%; respectively]. Figure 5 indicates periprocedural myocardial injury was most likely to occur in patients with higher maxLCBI4mm and greater PAV compared with those without (50.0% vs. 10.3% of frequency; OR 8.67; *p* < 0.001).

## Discussion

In the present study, we explored for the correlation between NIRS–IVUS-assessed coronary plaque characteristics and CCTA-derived characteristics as well as the incidence of periprocedural myocardial injury.

Our findings indicated that NIRS–IVUS-derived widespread LRPs were closely associated with CT density and the presence of napkin-ring sign by CCTA. Furthermore, the independent risk factors of periprocedural myocardial injury were maxLCBI4mm and PAV measured by NIRS–IVUS, not CCTA-derived factors.

### Correlation between NIRS–IVUS and CCTA for LRP detection

Periprocedural myocardial injury has been correlated to worse short- and long-term clinical outcome; therefore, various studies have previously identified high-risk plaques causing this “nightmare” by IVUS and CCTA (5, 17). Some studies have also revealed that the correlation between CCTA and gray-scale IVUS for LRP detection as well as consequently lower CT density was shown in the plaque with attenuation evaluated by IVUS (3, 18). Moreover, other CCTA studies have suggested that the napkin-ring sign was presented more frequently in high-risk plaques with a higher risk of ACS events (11).

A recent study showed that the group with a larger maxLCBI4mm had a smaller CT density and a higher proportion of napkin-ring signs (19). In addition, our results revealed that maxLCBI4mm has a linear inverse correlation with CT density.

### Predictive ability of NIRS-IVUS for periprocedural myocardial injury

Our study indicated that NIRS–IVUS-assessed maxLCBI4mm and PAV were significant risk factors for periprocedural myocardial injury. Previous studies clarified that the lesions with higher maxLCBI4mm were associated with a higher occurrence of periprocedural myocardial infarction (20, 21). Besides, previous optical coherence tomography study demonstrated that thin-cap fibroatheroma correlates well with lesions with large percentage PB, and an autopsy study clarified that the ability to identify fibroatheroma was significantly increased with the addition of NIRS-derived LCBI to gray-scale IVUS-derived PAV (22, 23). As a result of these findings, the combination of NIRS–IVUS-derived LRP and PAV might be a stronger predictor of periprocedural myocardial injury. In fact, our study revealed that the combination of increased plaque volume and a high maxLCBI4mm is a high-risk plaque with a higher incidence of periprocedural myocardial injury (Figure 5).

The NIRS-IVUS-derived maxLCBI4mm can only measure lipid distribution. Therefore, this fact also supports the hypothesis that the combination of maxLCBI4mm and PAV is useful for predicting periprocedural myocardial injury.

### Differences in predictive ability of periprocedural myocardial injury between NIRS-IVUS and CCTA

Our study showed that PAV and maxLCBI4mm are associated with periprocedural myocardial injury when PCI is performed with NIRS-IVUS in patients who have undergone prior CCTA. In other words, only PAV was a predictor of periprocedural myocardial injury in patients undergoing PCI with gray-scale IVUS alone.

Previous studies showed different values for the CT density threshold as the cutoff for LRP. Motoyama et al. demonstrated that a CT density of <30 HU indicated low-attenuation plaque according to the comparison of CCTA with gray-scale IVUS, whereas a pathological study revealed a cutoff value of low-attenuation plaque of <75 HU (10, 24).

In the case of CCTA, partial-volume effects due to the limitation of spatial resolution and motion artifacts caused by heartbeats and respiration could affect the measurement of CT density (10). Furthermore, if the lesion contains a calcified component, it may be difficult to assess the lumen and CT density by CCTA due to calcification artifacts (25). These features make it difficult to analyze plaque morphology as well as the volumetric measurement of the plaque in contact with the calcification. Moreover, this study focused on the culprit lesion, which has a large degree of stenosis, possibly making it difficult to accurately measure the lumen, especially with CCTA.

### Clinical implications

The PROSPECT2 study demonstrated that the high-risk plaques for non–culprit lesion-related MACE was maxLCBI4mm ≥324.7 and larger percent plaque area (9). In our study, a larger maxLCBI4mm and greater PAV were associated with periprocedural myocardial injury, indicating that patients with larger LRP and greater PAV are at higher risk for complications associated with PCI. This demonstrates that individuals with larger LRP and greater PAV are at high-risk not only for long-term prognosis for non-culprit plaques but also for perioperative complications associated with PCI. Our results suggest that assessing coronary plaque volume and plaque characteristics using NIRS–IVUS may be useful for predicting event in various situations, such as with both culprit and non-culprit lesions.

This study elucidated that CT density correlated well with maxLCBI4mm even in culprit lesions, implying that CCTA before PCI is useful for evaluating plaque characteristics. Recent research showed that aggressive lipid-lowering therapy (LLT) with statins and proprotein convertase subtilisin-kexin type 9 inhibitor has plaque-stabilizing and plaque-reducing effects on coronary plaque (26, 27). Therefore, more potent LLT options before PCI might be provided if CCTA reveals LRP in the target lesion. Subsequently, NIRS–IVUS can be used to evaluate plaque characteristics during PCI to determine the efficacy of preoperative drug treatment and to predict perioperative adverse events. Consequently, preoperative CCTA combined with NIRS–IVUS plaque assessment may contribute to further reduction of not only non–culprit lesion-related but also culprit lesion-related adverse events.

Accordingly, assessing maxLCBI4mm using NIRS-IVUS at the time of PCI can help predict perioperative complications, especially in patients who underwent CCTA prior to PCI and had low CT density in the culprit lesions.

### Study limitation

This study has several limitations. First, bias is possible due to the relatively small number of patients. A larger patient population is needed to confirm the results. Second, we excluded patients who were ineligible for CCTA before PCI (e.g., ST-elevation MI). Therefore, we could not assess all of the high-risk plaques in clinical practice. Third, patients in which imaging modality other than NIRS-IVUS was selected at the operator's discretion were excluded from this study. This may have involved the operator's selection bias. Fourth, primary prevention with oral medication before PCI might have affected the results. And finally, multivariable logistic regression was performed to analyze independent predictors for periprocedural myocardial injuries. Since the number of cases with periprocedural myocardial injury was relatively small in this study, it is possible that parameters other than those identified in this study may also play a role as the predictor of periprocedural myocardial injury.

## Conclusion

Although the NIRS–IVUS-derived LRP component was closely associated with low-attenuation plaque measured by CCTA, independent predictive factors for periprocedural myocardial injury were the NIRS–IVUS-derived larger PAV and higher maxLCBI4mm across the lesion. These were found to be the same predictors of coronary events in non-culprit lesions. The combination of pre-PCI plaque qualitative assessment by CCTA as culprit lesion screening and NIRS–IVUS in patients with suspected vulnerable plaques could identify and reduce the risk of periprocedural complications.

## Data Availability

The raw data supporting the conclusions of this article will be made available by the authors, without undue reservation.
