# Supplementary material for: Multimodality imaging to identify lipid-rich coronary plaques and predict periprocedural myocardial injury: Association between near-infrared spectroscopy and coronary computed tomography angiography
Source: Front Cardiovasc Med. 2023 Mar 30;10:1127121. doi: 10.3389/fcvm.2023.1127121 (PMC10108678; doi:10.3389/fcvm.2023.1127121)
Supplement: Supplementary file 1 [file Table1.docx]

Supplemental Table 1: Correlations between each predictors of periprocedural MI.

|  | QCA: Lesion length | CCTA: CT density | Gray-scale IVUS: PAV | NIRS-IVUS: maxLCBI4mm |
| --- | --- | --- | --- | --- |
| QCA: Lesion length |  | -0.156  (-0.341 to -0.041)  0.109 | 0.258  (0.066 to 0.432)  0.007 | 0.238  (0.045 to 0.414)  0.013 |
| CCTA: CT density |  |  | -0.248  (-0.423 to -0.055)  0.010 | -0.552  (-0.674 to -0.400)  <0.001 |
| Gray-scale IVUS: PAV |  |  |  | 0.261  (0.069 to 0.434)  0.007 |
| NIRS-IVUS: maxLCBI4mm |  |  |  |  |

Values are rho, (95% confidence interval), and *p*-value; from top to bottom for each column.

MI, myocardial infarction; QCA, quantitative coronary angiography; CCTA, coronary computed tomography angiography; IVUS, intravascular ultrasound; PAV, percent atheroma volume; NIRS, near-infrared spectroscopy; maxLCBI4mm, maximum value of the lipid core burden index for any 4-mm segment.
