# Supplementary material for: Multimodality imaging to identify lipid-rich coronary plaques and predict periprocedural myocardial injury: Association between near-infrared spectroscopy and coronary computed tomography angiography
Source: Front Cardiovasc Med. 2023 Mar 30;10:1127121. doi: 10.3389/fcvm.2023.1127121 (PMC10108678; doi:10.3389/fcvm.2023.1127121)
Supplement: Supplementary file 2 [file Presentation1.pdf]

SUPPLEMENTARY MATERIALS

Multimodality Imaging to Identify Lipid-rich Coronary Plaques and Predict Periprocedural Myocardial Injury: Association between Near-Infrared Spectroscopy and Coronary Computed Tomography Angiography

Supplemental Methods:

Definition details of the diagnosis of stable angina pectoris (SAP), silent myocardial ischemia (SMI), and non–ST-elevation acute coronary syndrome (NSTE-ACS)

SAP was defined as Canadian Cardiovascular Society (CCS) class 1 or 2. SMI was defined as no symptoms of angina pectoris for at least 6 weeks prior to percutaneous coronary intervention (PCI) but a positive functional test for ischemia within 30 days before PCI. In contrast, NSTE-ACS included patients with CCS class 3 or 4 ischemic chest discomfort without ST segment elevation on electrocardiogram (ECG). All patients showed no elevation in cardiac troponin T (cTnT; <0.016 ng/mL: institutional upper limit of normal) before PCI or stable cTnT levels (≤20% variation) or falling levels in patients with baseline cTnT elevation. In this study, patients with cTnT > institutional upper normal limits before PCI were defined as NSTEMI, while those who did not were defined as unstable AP. To distinguish between

PCI-induced cTnT elevation and the index ACS, we identified patients in whom cTnTs were stable or falling after admission to PCI.

Coronary Computed Tomography Angiography (CCTA) measurement protocol

The technique of CCTA measurement and analysis has been previously reported (1). ECG-gated CTA was performed using SOMATOM Definition AS+ (Siemens Healthcare, Forchheim, Germany) within 90 days before coronary angiogram (CAG). Iopamiron 370 mg iodine/mL (Bayer Health Care, Osaka, Japan) or Omnipaque 350 mg iodine/mL (Daiichi Sankyo, Tokyo, Japan) were used in a contrast-enhanced ECG gating scan to collect CCTA data.

Prior to the scan, beta-blockers were administered orally and intravenously to the patient to reduce heart rate. Following sublingual nitroglycerin spray (0.6 mg) administration, a prospective ECG-gated helical scan was performed with the following parameters: collimation width of 2 × 64 × 0.6 mm, rotation time of 300 ms/r, tube voltage of 100 kV for 64 thin patients (body mass index [BMI] <18.5 kg/m^2), and 120 kV for 43 normal patients (18.5 ≤ BMI ≥ 25.0 kg/m^2) or obese patients (BMI > 25.0 kg/m^2), effective tube current 300 to 400 mAs, table feed 11.5 mm/rotation, and pitch 0.18 to 0.20. Using a slice thickness of 0.75 mm and an increment of 0.4 mm, raw CT data were reconstructed. Individually, the best cardiac phase with the least amount of motion artifact was identified.

Experienced technicians blinded to quantitative coronary angiography (QCA) and near-infrared spectroscopy intravascular ultrasound (NIRS-IVUS) analyzed the scans using the Aquarius iNtuition edition version 4.4.8 (TeraRecon, Frankfurt, Germany) three-dimensional workstation. Cross-sectional images were used to evaluate the outer vessel area and the remodeling index (RI) of the vessel. The RI was calculated as the EEM cross-sectional area (CSA) of the target lesion divided by the average of the EEM-CSAs of the proximal and distal reference segments; positive remodeling was defined as a RI of ≥1.10 (2).

Preoperative antiplatelet therapy and perioperative anticoagulant therapy

All patients received dual antiplatelet medication before the procedure, which included oral administration of aspirin (81–100 mg) and clopidogrel (75 mg daily or 300-mg loading dose) or prasugrel (3.75 mg daily or 20-mg loading dose). All patients also received an intravenous bolus injection of 8000–10000 IU heparin, and an additional bolus of 2000–3000 IU was given every hour if the procedure lasted for more than 1 hour to maintain an activated clotting time >300 seconds.

QCA Analysis

Standard image acquisition was performed with at least two orthogonal projections of the lesion stenosis in CAG, preceded by an intracoronary injection of nitroglycerin to maximally dilate the coronary artery vessels.

An experienced technician blinded to CCTA, IVUS, and NIRS data interpreted the images using a validated automated edge-detection program (CCIP-310/W, Cathex, Tokyo, Japan). Morphological variables such as lesion calcification, defined as radio-opacities through the vessel wall, and thrombus-containing lesions, defined as spherical filling defects surrounded by contrast observed in multiple projections were collected. QCA was preceded by system calibration using the external diameter of the catheter filled with contrast medium. Subsequently, the minimal lumen diameter and reference segment diameter were measured at the end of diastole, and the result of the "worst" view were recorded.

Gray-scale IVUS and NIRS analyses and chemometrics

Details of the NIRS-IVUS system and analysis have been described previously (3, 4). All IVUS analyses were performed with the catheter appropriately advanced distally following CAG or initial PCI. An automatic mechanical pullback (TVC-MC8, 0.5 mm/s, 960 rpm; TVC-MC10, 0.5 mm/s, 1800 rpm) was used to bring the IVUS catheter back to the aorto-ostial junction at a speed of 0.5 mm/s. The system demonstrates grayscale IVUS images and color-coded NIRS data in a single pullback at the same time.

The probability of lipid core plaque (LCP) during catheter pull-back was indicated by a digital color-coded map "chemogram" that includes the location and intensity of the LCP. Spectral information at each pixel was converted to a probability of LCP and then mapped onto a 128 (7-bit) red-to-yellow color scale, with low lipid content probabilities indicated by red and high probabilities by yellow. The maximum lipid core burden index (LCBI) was calculated to provide a quantitative summary metric of the LCP across the entire scanned segment. The LCBI is the fraction of valid pixels in the chemogram of >0.6 for LCP, multiplied by 1000. Using the top 10th percentile pixel information from the corresponding 2-mm NIRS segment, the block chemogram exhibited colored blocks according to the probability of identifying a lipid plaque. If the top 10th percentile probability value for lipids was ≥0.98, the block was displayed in yellow; 0.84–0.97 in tan; 0.57–0.84 in orange; and <0.57 in red. The maxLCBI4mm was defined as the maximum value of the LCBI for any 4-mm segment in the target lesion, including the minimal lumen area site.

References

1. Imai S, Kondo T, Stone GW, Kawase Y, Ahmadi AA, Narula J, et al. Abnormal Fractional Flow Reserve in Nonobstructive Coronary Artery Disease. *Circ Cardiovasc Interv*. (2019) 12:e006961. doi: 10.1161/CIRCINTERVENTIONS.118.006961
2. Motoyama S, Kondo T, Anno H, Sugiura A, Ito Y, Mori K, et al. Atherosclerotic plaque characterization by 0.5-mm-slice multislice computed tomographic imaging. *Circ J*. (2007) 71:363-366. doi: 10.1253/circj.71.363
3. Ota H, Magalhaes MA, Torguson R, Negi S, Kollmer MR, Spad MA, et al. The influence of lipid-containing plaque composition assessed by near-infrared spectroscopy on coronary lesion remodelling. *Eur Heart J Cardiovasc Imaging*. (2016) 17:821-31. doi: 10.1093/ehjci/jev221
4. Ota H, Omori H, Kawasaki M, Hirakawa A, Matsuo H. Clinical impact of PCSK9 inhibitor on stabilization and regression of lipid-rich coronary plaques: a near-infrared spectroscopy study. *Eur Heart J Cardiovasc Imaging*. (2022) 23:217-28. doi: 10.1093/ehjci/jeab034
